# Cigarette Smoke Increases CD8α^+^ Dendritic Cells in an Ovalbumin-Induced Airway Inflammation

**DOI:** 10.3389/fimmu.2017.00718

**Published:** 2017-06-16

**Authors:** Thayse Regina Brüggemann, Paula Fernandes, Luana de Mendonça Oliveira, Maria Notomi Sato, Mílton de Arruda Martins, Fernanda Magalhães Arantes-Costa

**Affiliations:** ^1^Laboratory of Experimental Therapeutics LIM20, Department of Medicine, School of Medicine, University of Sao Paulo, Sao Paulo, Brazil; ^2^Pulmonary and Critical Care Medicine, Department of Medicine, Brigham and Women’s Hospital, Boston, MA, United States; ^3^Laboratory of Medical Investigation LIM56, School of Medicine, Division of Clinical Dermatology, University of Sao Paulo, Sao Paulo, Brazil

**Keywords:** mouse model, asthma, cigarette smoke, dendritic cells, CD4^+^ T cells, CD8^+^ T cells

## Abstract

Asthma is an allergic lung disease and, when associated to cigarette smoke exposition, some patients show controversial signs about lung function and other inflammatory mediators. Epidemiologic and experimental studies have shown both increasing and decreasing inflammation in lungs of subjects with asthma and exposed to cigarette smoke. Therefore, in this study, we analyzed how cigarette smoke affects pro-inflammatory and anti-inflammatory mediators in a murine model of allergic pulmonary inflammation. We sensitized Balb/c mice to ovalbumin (OVA) with two intraperitoneal injections. After sensitization, the animals were exposed to cigarette smoke twice a day, 30 min per exposition, for 12 consecutive days. In order to drive the cell to the lungs, four aerosol challenges were performed every 48 h with the same allergen of sensitization. OVA sensitization and challenge developed pulmonary Th2 characteristic response with increased airway responsiveness, remodeling, increased levels of IgE, interleukin (IL)-4, and IL-13. Cigarette smoke, unexpectedly, reduced the levels of IL-4 and IL-13 and simultaneously decreased anti-inflammatory cytokines as IL-10 and transforming growth factor (TGF)-β in sensitized and challenged animals. OVA combined with cigarette smoke exposition decreased the number of eosinophils in bronchoalveolar lavage and increased the number of neutrophils in lung. The combination of cigarette smoke and lung allergy increased recruitment of lymphoid dendritic cells (DCs) into lymph nodes, which may be the leading cause to an increase in number and activation of CD8^+^ T cells in lungs. In addition, lung allergy and cigarette smoke exposure decreased an important regulatory subtype of DC such as plasmacytoid DC as well as its activation by expression of CD86, PDL2, and ICOSL, and it was sufficient to decrease T regs influx and anti-inflammatory cytokines release such as IL-10 and TGF-β but not enough to diminish the structural changes. In conclusion, we observed, in this model, that OVA sensitization and challenge combined with cigarette smoke exposure leads to mischaracterization of the Th2 response of asthma by decreasing the number of eosinophils, IL-4, and IL-13 and increasing number of neutrophils, which is related to the increased number of CD8ɑ^+^ DCs and CD8^+^ T cells as well as reduction of the regulatory cells and its released cytokines.

## Introduction

Asthma is among the most prominent and severe types of allergic diseases, affecting 300 million people worldwide (1). The pulmonary response to allergens is initiated by the activated epithelium-releasing TSLP, interleukin (IL)-25, and IL-33 that consequently activate innate immune cells such as ILC2s. Once activated, ILC2s release IL-4 and IL-13 that are main keys to the inflammatory features on asthma (2). The environment created by inflammatory cytokines and inflammatory cells activate the adaptive immune system characteristically denominated as a persistent Th2 inflammation. Th2 response is dominated by the presence of eosinophils, IgE, and cytokines such as IL-4 and IL-13 (3–5). As a counter regulatory response, regulatory T cells (Tregs) are recruited to the site of inflammation and released cytokine such as IL-10. This cytokine is often but not always capable of attenuating Th2 response in some models of lung allergy and; therefore, it plays a protective role in allergic asthma (6).

The way that adaptive immune response is generated depends on dendritic cells (DCs) interaction with the antigen in the airways and consequently generation of proinflammatory or regulatory response by expressing different markers on its surface (7, 8). Per each marker expressed by DCs, they can be differentiated phenotypically and functionally. CD11b^+^ DCs are the most abundant common DCs (cDCs) in lymphoid organs except for the thymus and can also be found in non-lymphoid tissues (9). In a murine model of pulmonary allergic inflammation, cDCs play crucial roles in initiating and maintaining the immune response (10). CD8α^+^ DC can be found in murine lymphoid organs and sometimes can play a regulatory role in asthma by enhanced cross-priming CD8^+^ T cells [reviewed in Ref. (9)]. Plasmacytoid DCs (pDCs) are known to play a regulatory role in asthma by releasing IL-10 (11).

It is being showed that active smoking interacts with the asthmatic phenotype, causing more severe allergic symptoms, a greater decline in lung function, and impaired therapeutic responses to corticosteroids (12–14). In addition, smoking asthmatics are admitted to hospitals with asthma-related exacerbations at a greater rate than asthmatics that do not smoke (12). Nevertheless, animal models have showed that mainstream cigarette smoke has the potential to break primary inhalational tolerance to allergens in naive animals and to increase the systemic sensitization to surrogate and environmental allergens (15, 16) by direct toxicity, oxidative damage, the recruitment of inflammatory cells, and increased epithelial permeability (17). Smoking is associated with contradictory both release and inhibition of pro-inflammatory and anti-inflammatory mediators (18), but one outstanding observation was that the effects of smoke exposure on allergic responses appear to be dose dependent (19).

To further explore the role of cigarette smoke exposure on allergic airway inflammation, we sensitized mice to the allergen ovalbumin (OVA) and then exposed them to cigarette smoke both before and after challenge with inhaled OVA aerosol. We asked ourselves how cigarette smoke affects the adaptive immune response in this model of asthma. We report here that cigarette smoke acts in an allergic lung disease reducing proinflammatory cytokines but not in a way that diminished inflammation. The mechanism involved in this changing of the Th2 response is related to the increased recruitment of lymphoid DCs (CD8ɑ^+^ DCs) that play a regulatory pathway in asthma, but are not sufficient to diminish the structural changes.

## Materials and Methods

### Animals

The ethics Committee of the School of Medicine of University of Sao Paulo approved all the experiments. Male BALB/c mice (8 weeks old) were purchased from the University of Sao Paulo (Sao Paulo, Brazil) and maintained as described in the Guide for the Care and Use of Laboratory Animals—NIH (20). Mice were divided into four groups: SAL (non-sensitized and non-exposed to cigarette smoke), CS (exposed to cigarette smoke), OVA (OVA sensitized and challenged), and O + CS (OVA sensitized, challenged, and exposed to cigarette smoke).

### Sensitization Protocol

**Figure 1:**
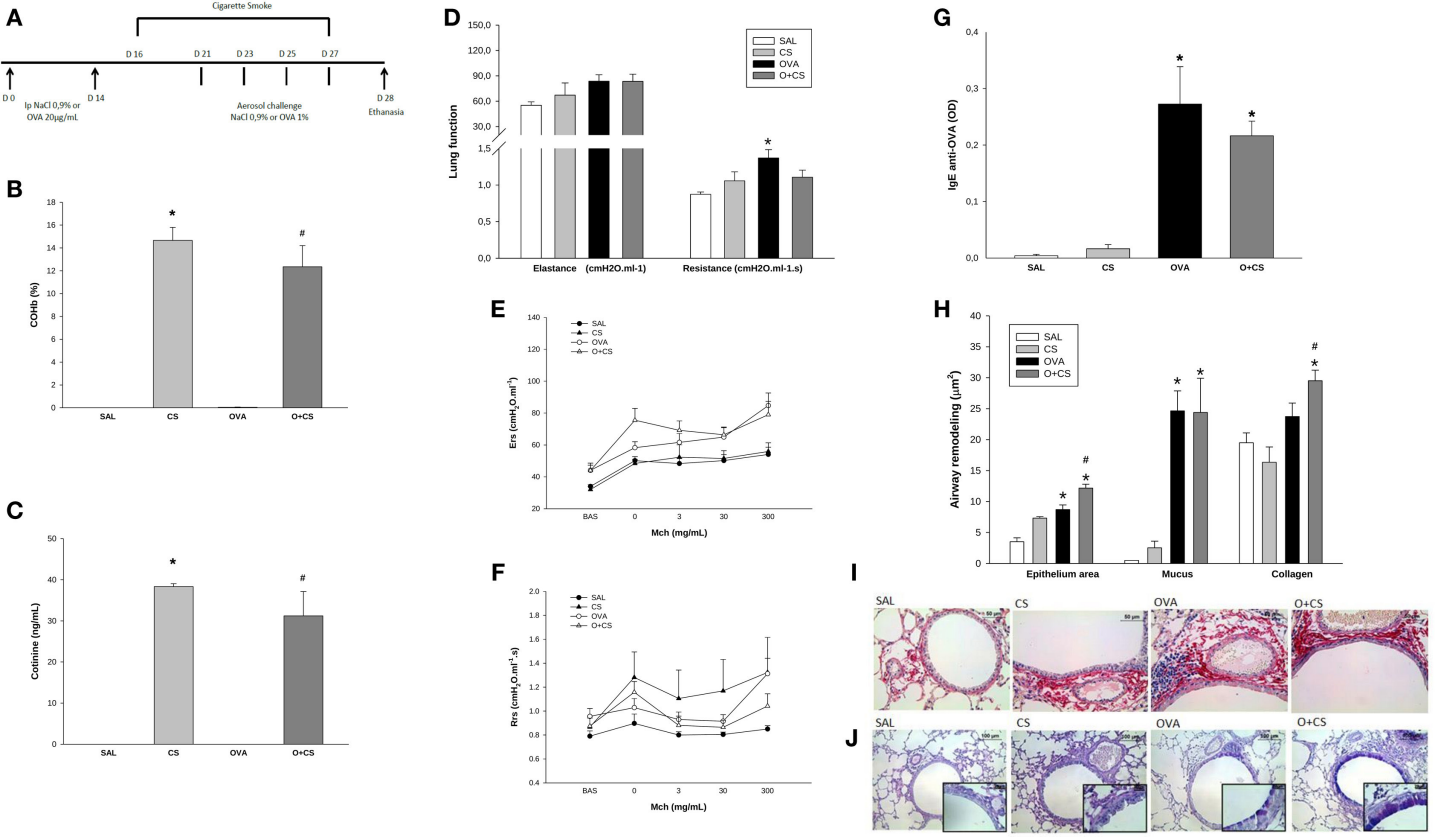
Time line of experimental protocol **(A)**: sensitization with ovalbumin (OVA)/NaCl on days 0 and 14. Cigarette smoke exposure from day 16 to 27. Aerosol challenge with OVA/NaCl on days 21, 23, 25, and 27. Harvest on day 28; results are presented as mean ± SE; carboxyhemoglobin (COHb) levels in blood **(B)**; *n* = 4 mice/group; *p < 0.001 when compared CS versus SAL; ^#^*p* = 0.001 when compared O + CS versus OVA; cotinine levels in serum **(C)**; *n* = 4 mice/group; *p < 0.001 when compared CS versus SAL; ^#^*p* = 0.001 when compared O + CS versus OVA; maximal response of elastance and resistance of respiratory system **(D)**; *n* = 5–0 mice/group; *p < 0.006 when compared OVA versus SAL; methacholine dose–response curve values of elastance **(E)**; methacholine dose–response curve values of resistance **(F)**; serum IgE production **(G)**; *n* = 5–10 mice/group; *p < 0.001 when compared OVA versus SAL and *p* = 0.008 when compared O + CS versus CS; airway remodeling presented as epithelium area, mucus area, and collagen fibers **(H)**: epithelium: *n* = 5–7 mice/group; *p = 0.004 when compared OVA versus SAL and *p* < 0.001 when compared O + CS versus CS; ^#^*p* = 0.01 when compared O + CS versus OVA; mucus area: *n* = 5–7 mice/group; *p < 0.001 when compared OVA versus SAL or when compared O + CS versus CS; collagen: *n* = 5–10 mice/group; *p < 0.001 when compared O + CS versus CS; ^#^*p* < 0.001 compared O + CS versus OVA; representative photomicrographs of collagen fibers stained with picrosirius in all groups **(I)**; representative photomicrographs of epithelium remodeling and mucus deposition on airways stained with PAS-AB in all groups **(J)**.

Animals from OVA and O + CS groups were sensitized with two intraperitoneal injections containing 20 µg/mL of OVA (Grade V, Sigma Chemical Co., St. Louis, MO, USA) adsorbed in 3 mg/mL of aluminum hydroxide (Pepsamar gel, Sanofi-Synthelabo S.A., Rio de Janeiro, Brazil) (21). Animals received the intraperitoneal injections of OVA in a total volume of 0.2 mL on days 0 and 14. Animals from SAL and CS groups received two intraperitoneal injection containing saline solution (NaCl 0.9%) and aluminum hydroxide on same days. On days 21, 23, 25, and 27, the animals were challenged *via* aerosol for 30 min. OVA sensitized groups were challenged with OVA solution at 1% and non-sensitized groups were challenged with saline solution. On day 28, the animals were studied (Figure 1A).

### Cigarette Smoke Exposition Protocol

The CS and O + CS groups were placed inside a box divided in eight sections. The box was connected to a Venturi system that drove the cigarette smoke directly into the box, mimetizing a first-hand smoke system. Animals were exposed to seven commercial cigarettes (Derby Filter Red, 0.8 mg of nicotine, 10 mg of tar, and 10 mg of CO per cigarette, Souza Cruz, UDI, Brazil) without filters twice per day for 30 min (22) during 12 consecutive days from day 16 to 27 (Figure 1A). The concentration of CO inside the box was maintained between 350 and 400 ppm during entire time of exposition. The interval between each exposition was of 3 h. The aerosol challenge was performed 1 h after the first cigarette smoke exposition so it would not affect the antigen presentation.

### Cotinine and Carboxyhemoglobin (COHb) Analysis

To evaluate COHb levels in blood and to see if sensitization and challenge would interfere in these levels, mice (four per group) were euthanized right after last exposure to cigarette smoke (day 28 of the protocol) and 0.2 mL of blood was collected. Levels of COHb were obtained by blood gas analyzer (ABL800, Radiometer, CA, USA). The quantification of cotinine levels in blood was evaluated using a cotinine ELISA kit as manufacturer instructions (Abnova, CA, USA).

### Airway Responsiveness Evaluation

Twenty-four hours after the last challenge and cigarette smoke exposition, we evaluated airway responsiveness of eight to ten mice per group, in a whole-body plethysmograph system (Harvard Apparatus, Boston, MA, USA). After the baseline reading, anesthetized (thiopental 33 mg, 0.01 mL/g), tracheostomized, and ventilated mice were serially exposed to increasing concentrations of nebulized methacholine (Mch) at 3, 30, and 300 mg/mL for 1 min per dose. The values of elastance and resistance were captured after each dose of Mch at 30 s, 1, 2, and 3 min (21). The highest dose, regardless of the time, was chosen to determine maximal respiratory response.

### Specific Antigen-Antibody and Cytokines Analysis

Right after the airway responsiveness evaluation, 0.3 mL of blood from five to ten mice per group, was collected and diluted into 600 µL of saline solution. Then it was centrifuged at 3,000 rpm for 10 min at 4°C. The serum was maintained at −20°C until IgE analysis. ELISA kit was used to quantify serum and IgE levels according to manufacture instructions (R&D Systems, MN, USA). The levels of IL-4, IL-10, IL-13, and transforming growth factor (TGF)-β1 in the lung homogenate was measured using ELISA kit according to manufacture instructions (R&D Systems, MN, USA).

### Lung Histology Analysis

Left lungs of five to ten mice per group were fixed in 10% formalin and embedded in paraffin. Five-micrometer-thick sections were used to make histology slides. Lung histology slides were stained for periodic acid-Schiff-alcian blue (PAS-AB) in order to quantify the area of the bronchial epithelium area and mucus. The evaluation was made using an optical microscope with one of the oculars designed with a square with 50 lines and 100 dots and with a known area of 10,000 µm^2^. The mucus and epithelium area was measured by counting the number of dots that reached the mucus or epithelium respectively. The area was calculated knowing that 100 dots are equivalent of 10,000 µm^2^, and then the number of dots that reached mucus/epithelium would be equivalent of the area in square micrometer. In order to quantify collagen fibers, histology slides with lung slices were stained with picrossirius red. Using a microscope with camera, pictures of four airways per slide were taken and analyzed for the area in square micrometer of collagen fibers using Image Pro Plus software (Media Cybernetics Inc., MA, USA) (21, 23).

### Cell Phenotype

Mice (eight per group) were euthanized and bronchoalveolar lavage (BAL) fluid was performed. Lungs were washed with 1.5 mL of saline solution, then the volume was centrifuged at 1,500 rpm for 10 min at 4°C. The pellet of cells was resuspended with 1 mL of saline and total number of cell was counted using a hemocytometer. After BAL, lungs were perfused with 5 mL of saline. Then, lungs were harvested and cut in small pieces with scissors, incubated in solution containing collagenase (Sigma-Aldrich, 0.7 mg/mL) and DNase1 (Sigma-Aldrich, 30 µg/mL) for 30 min at 37°C. After incubation, suspension cells were filtered with 40 µm strainer and the enzymes were blocked with PBS added of serum at least three times of the initial volume. The cell suspension was centrifuged at 1,500 rpm for 10 min and resuspended in 1 mL of saline for total cell counting. Lymph nodes were harvested and homogenized using saline solution and a 40 µm strainer. The volume was centrifuged and the pellet with cells was resuspended with 1 mL of saline for total cell counting. Cells were stained for cell surface and intracellular markers. The phenotype of the cells was evaluated using the following antibodies: anti-B220-PE-CY7 (RA3-6B2), anti-FOXP3-V450 (MF23), anti-LY6C and G-APC-CY7 (RB6-8C5), anti-MHC II-PE or V500 (M5/114.15.2 and M5115.15.2, respectively), anti-SIGLEC-F-PE-CF594 (E50-2440), anti-CD3-PERCP (145-2C11), anti-CD4-V500 (RM4-5), anti-CD8α-APC-Cy7 (53-6.7), anti-CD11b-PE-CY7 or BV605 (M1/70), anti-CD11c-FITC (HL3), anti-CD24-PERCP-CY5.5 (M1/69), anti-CD25-PE-CY7 (PC61), anti-CD69-FITC (H1.2F3), anti-CD86-Alexa Fluor 700 (GL1), anti-ICOSL-PE (hk5.3), and PDL2-APC (Ty25), all of which were purchased from BD Bioscience, NJ, USA. F4/80-eFluor-450 (BM8) and PDCA1-eFluor-450 (eBio927) were purchased from eBioscience, SD, USA. A total of 1 × 10^5^ live events was acquired and analyzed with flow cytometry (LSR Fortessa, BD, San Jose, CA, USA) and FlowJo 10.0.6 (Tree Star, Ashland, OR, USA). Fluorescence minus one controls were performed for all antibody panels. The analysis strategy is provided in Figures S1–S7 in Supplementary Material.

### Statistical Analysis

Statistical differences between experimental groups were detected by analysis of variance (two-way ANOVA) followed by the Holm–Sidak *post hoc* test for multiple comparisons (SigmaStat 2.03, SPSS, Chicago, IL, USA). *p*-Values <0.05 were considered significant.

## Results

### Cigarette Smoke Exposure Increases Remodeling in OVA-Sensitized Animals

We analyzed COHb and cotinine levels, and the levels of both substances were comparable to those observed in a heavy smoker (Figures 1B,C) (24, 25).

We next analyzed whether the OVA protocol was able to stimulate an inflammatory process similar to human asthma. As we found in this model, OVA sensitization and aerosol challenge causes pulmonary allergic inflammation that is similar asthma in humans and characterized by higher lung remodeling (Figure 1H) and an increase in IgE levels (Figure 1G). We also observed hyperresponsiveness in the OVA group with increased maximal response of resistance (Figure 1D). However, there was no difference of resistance or elastance among different doses of methacholine among all groups (Figures 1E,F). We still observed that the combination of cigarette smoke exposure in this model of asthma caused higher deposition of collagen fibers over the airway when compared to CS alone and OVA group and promoted hyperplasia/hypertrophy of bronchial epithelial cells (Figures 1H–J).

### Cigarette Smoke Exposure Decreases Pro-inflammatory and Anti-inflammatory Cytokines in OVA-Sensitized Animals

**Figure 2:**
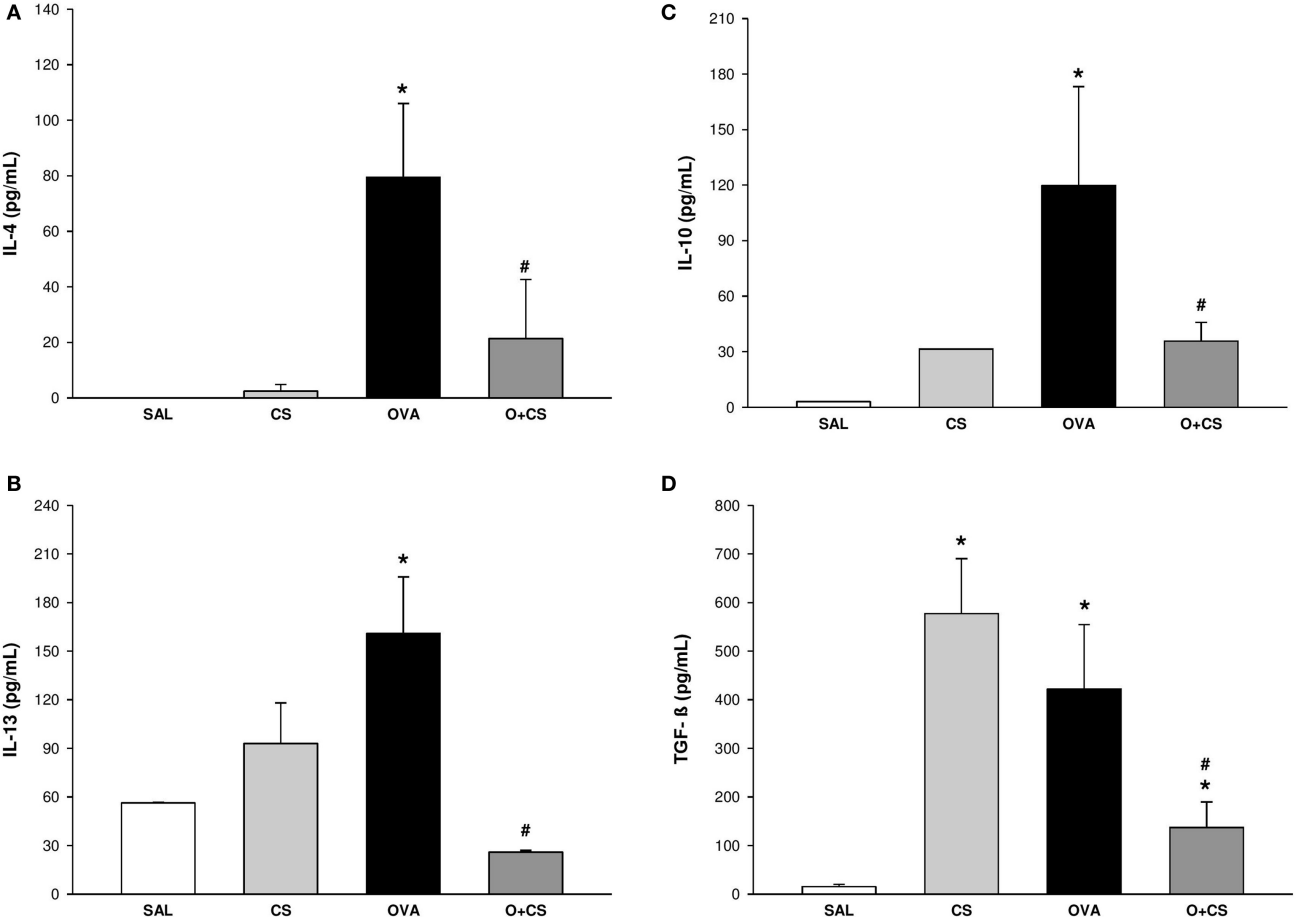
Cytokines levels in lung homogenate. Results are presented as mean ± SE; *n* = 5–10 mice/group; interleukin (IL)-4. **(A)** *p = 0.009 when compared ovalbumin (OVA) versus SAL; ^#^*p* = 0.029 when compared O + CS versus OVA; IL-13. **(B)** *p = 0.043 when compared OVA versus SAL; ^#^*p* = 0.026 when compared O + CS versus OVA; IL-10. **(C)** *p < 0.001 when compared OVA versus SAL; ^#^*p* = 0.005 when compared O + CS versus OVA; transforming growth factor (TGF)-β. **(D)** *p < 0.001 when compared CS versus SAL, *p* = 0.005 when compared OVA versus SAL and *p* = 0.002 when compared O + CS versus CS; ^#^*p* = 0.029 when compared O + CS versus OVA.

We investigated the Th2 adaptive immune response, which has the characteristic cytokines of the allergic inflammatory response of the model we used. For that, we quantified IL-4, which increased in the OVA group but decreased significantly in the O + CS group (Figure 2A). To clarify the possible mechanisms involved in the increased responsiveness and remodeling observed before, we analyzed IL-13, which showed an increased level in the OVA group and interestingly decreased in the O + CS group (Figure 2B). We also analyzed IL-10 and TGF-β, which are present in inflammation as regulatory cytokines. The OVA group presented higher levels of IL-10 and TGF-β than the SAL group. Both of these cytokines decreased when OVA was combined with CS (Figures 2C,D) leading to an inflammatory profile. No significant differences were found in IFN-γ levels among the groups (data not shown).

### Cigarette Smoke Exposition Leads to Decrease of Eosinophils but Increase in Neutrophils

**Figure 3:**
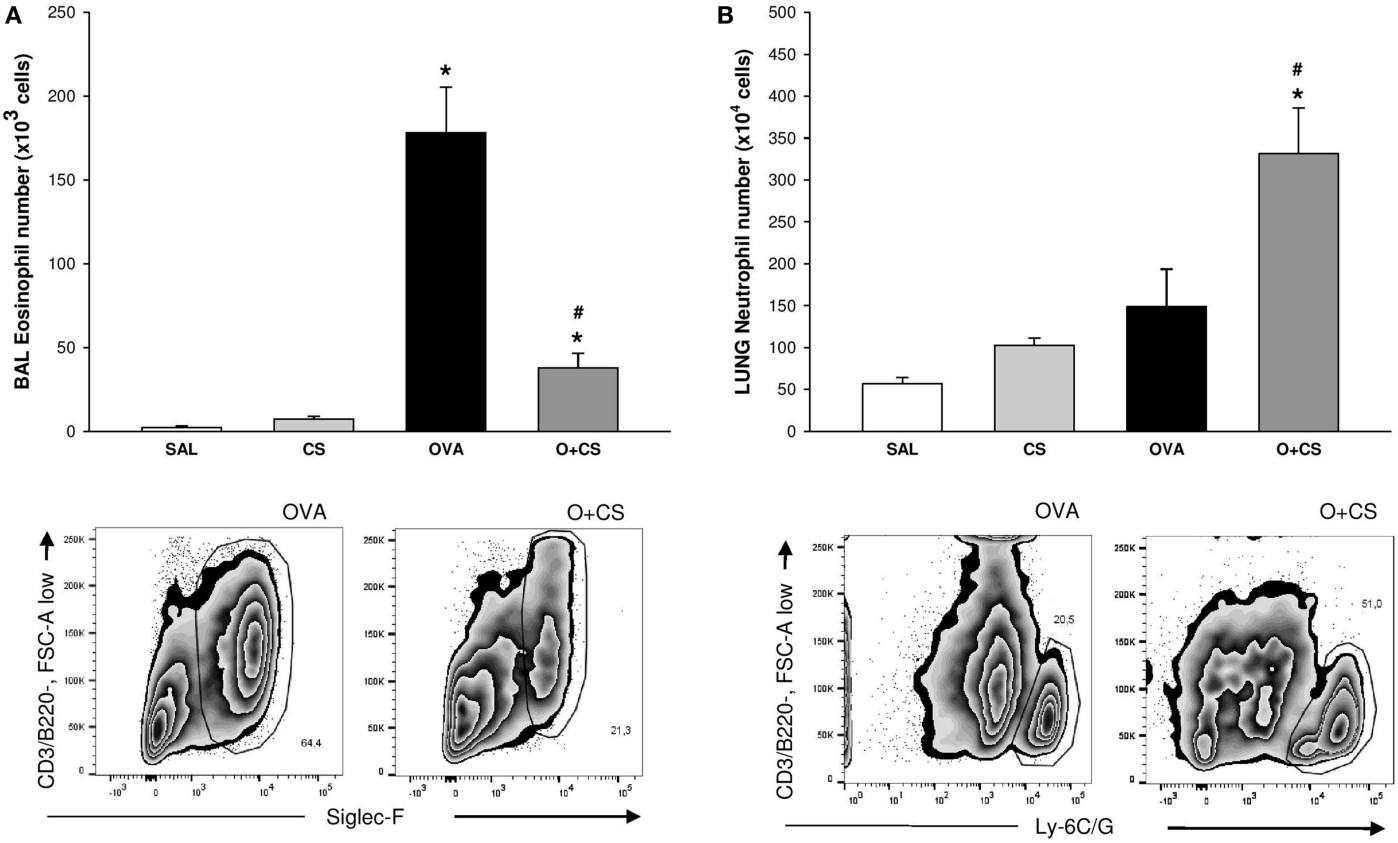
Number of eosinophils in bronchoalveolar lavage (BAL) and number of neutrophils in lung tissue. Results are presented as mean ± SE; *n* = 8 mice/group; eosinophils. **(A)** *p < 0.001 when compared ovalbumin (OVA) versus SAL and *p* = 0.041 when compared O + CS versus CS; ^#^*p* < 0.001 when compared O + CS versus OVA; neutrophils. **(B)** *p < 0.001 when compared OVA versus SAL; ^#^*p* = 0.003 when compared O + CS versus OVA.

We observed, in the OVA group, an increased number of eosinophils (Figure 3A). When OVA allergy is combined with CS exposure, the number of eosinophils in BAL decreased compared to OVA group (Figure 3A), whereas the number of neutrophils significantly increased in lung tissue (Figure 3B), indicating a switch in asthma phenotype inflammation.

### Type 2 Macrophages Increase When OVA and Cigarette Smoke Exposure Are Combined

**Figure 4:**
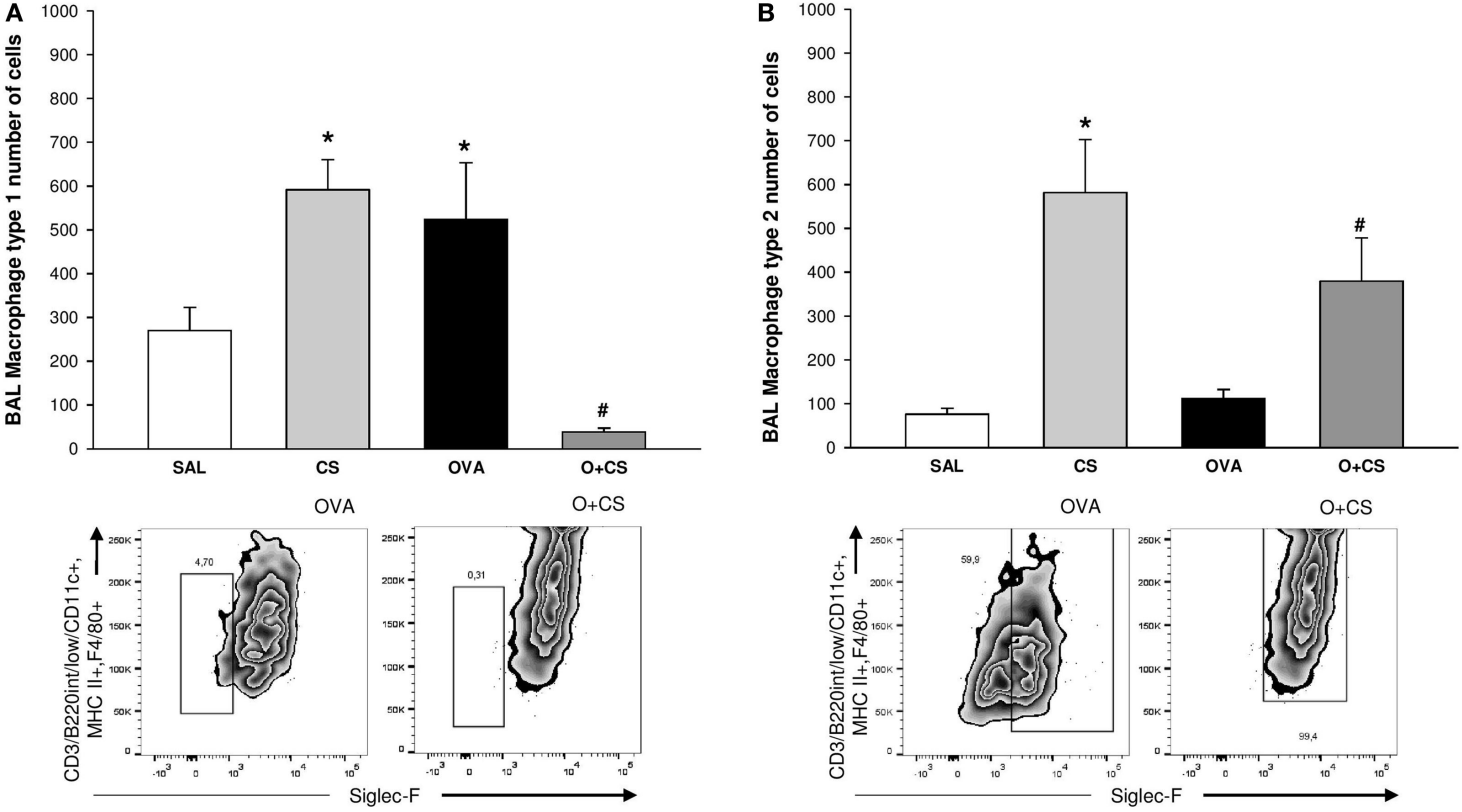
Type 1 macrophage (M1) and type 2 macrophage **(B)** in bronchoalveolar lavage (BAL). Results are presented as mean ± SE; *n* = 8 mice/group; M1. **(A)** *p = 0.005 when compared CS versus SAL, *p* = 0.027 when compared ovalbumin (OVA) versus SAL and *p* < 0.001 when compared O + CS versus CS; ^#^*p* < 0.001 when compared O + CS versus OVA; M2. **(B)** *p < 0.001 when compared CS versus SAL; ^#^*p* = 0.012 when compared O + CS versus OVA.

Macrophages are the most abundant immune cell present in the lung, and these cells have important functions in lung homeostasis such as responding to infection and inflammation (26). We observed both types, M1 and M2, of macrophages in the BAL, and we found that cigarette smoke alone was capable of increasing both of them. Although OVA alone only increased the M1 macrophages, interestingly, in the O + CS group, we found a decrease in M1 macrophages but a substantial increase in M2 macrophages relative to OVA (Figures 4A,B).

### Lung CD8^+^ T Cells Are Recruited and Activated by CS Exposure in OVA-Sensitized Animals

**Figure 5:**
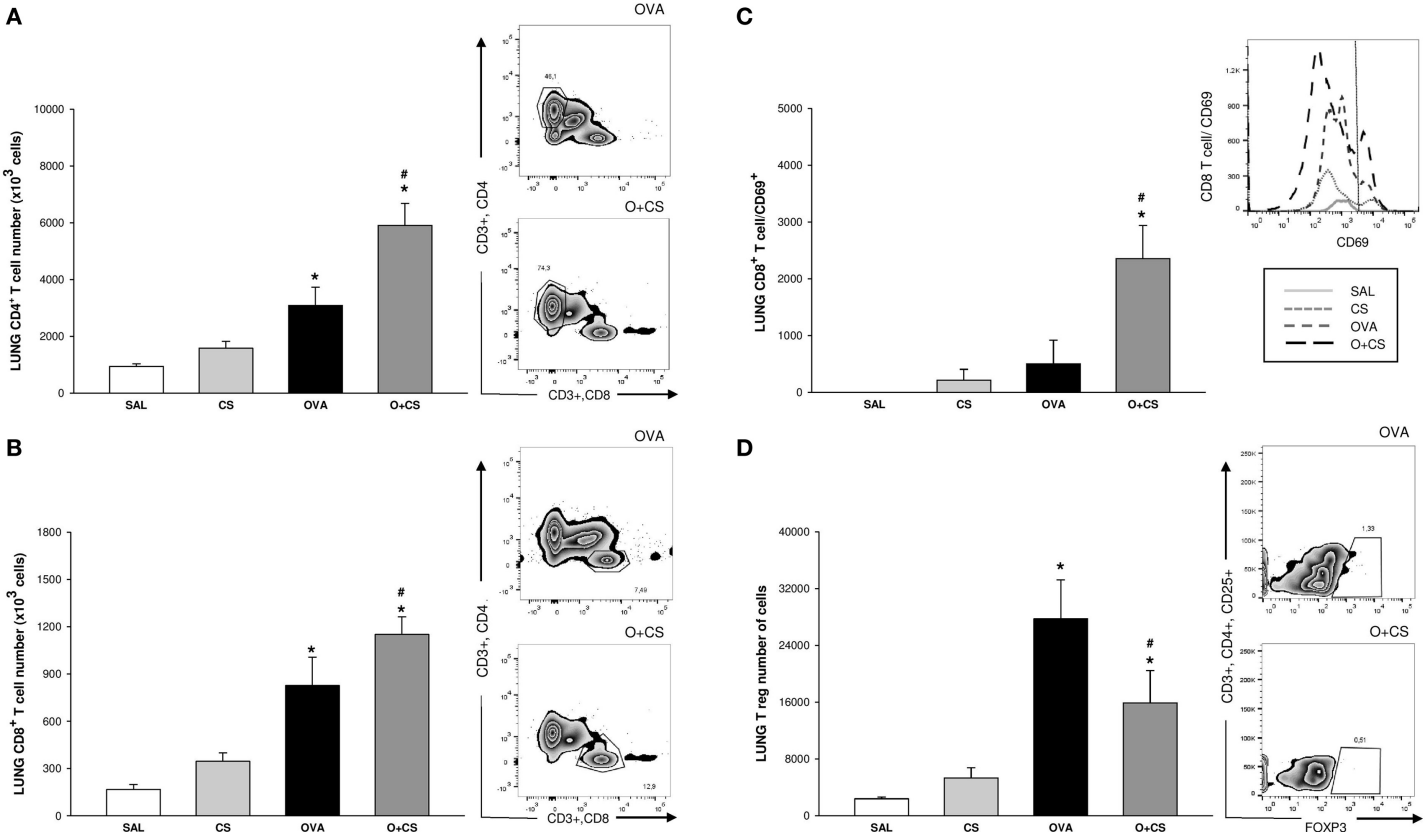
Number of CD4^+^ T cell, CD8^+^ T cell, MFI of CD69 CD8^+^ T cells, and number of T reg cells in lung. Results are presented as mean ± SE; *n* = 8 mice/group; CD4^+^ T cell. **(A)** *p < 0.001 when compared ovalbumin (OVA) versus SAL or O + CS versus CS. ^#^*p* = 0.05 when compared O + CS versus OVA; CD8^+^ T cell. **(B)** *p < 0.001 when compared OVA versus SAL or O + CS versus CS; ^#^*p* = 0.048 when compared O + CS versus OVA; MFI of CD69 CD8^+^ T cell. **(C)** *p < 0.001 when compared O + CS versus CS; ^#^*p* < 0.001 when compared O + CS versus OVA; T regs. **(D)** *p < 0.001 when compared OVA versus SAL and *p* = 0.043 when compared O + CS versus CS; ^#^*p* = 0.031 when compared O + CS versus OVA.

To identify what was responsible for the decrease in cytokine levels, we analyzed the number of lymphocytes and their activation. OVA sensitization increased both CD4^+^ and CD8^+^ T cells in the lung tissue. Exposure to OVA and CS significantly intensified this OVA-induced increase in both CD4^+^ and CD8^+^ T cells in addition to activating CD8^+^ T cells (Figures 5A–C). T reg cells increased with OVA sensitization, and a drastic decrease was observed when sensitization was combined with CS exposure (Figure 5D).

### DCs Are Activated in OVA-Sensitized Animals by CS Exposure

**Figure 6:**
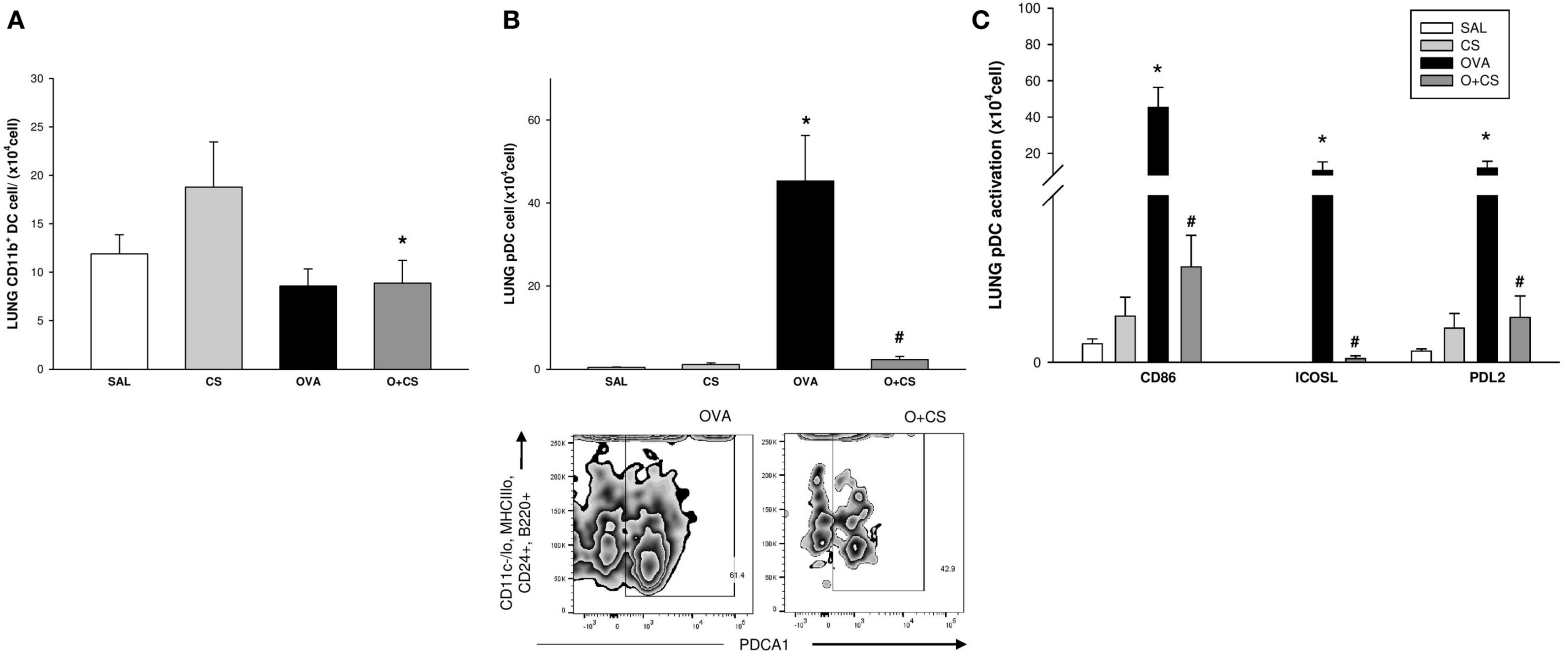
Number of CD11b + dendritic cells (DCs), plasmacytoid DCs (pDCs), and pDCs activation by expression of CD86, ICOSL, and PDL2in lung; results are presented as mean ± SE; *n* = 8 mice/group; CD11b^+^ DCs. **(A)** *p = 0.017 when compared O + CS versus CS; pDCs. **(B)** *p < 0.001 when compared ovalbumin (OVA) to SAL; ^#^ *p* < 0.001 when compared O + CS to CS; expression of CD86, ICOSL, and PDL2 by pDCs. **(C)** *p ≤ 0.005 when compared O + CS versus CS; ^#^*p* ≤ 0.004 when compared O + CS versus OVA.

**Figure 7:**
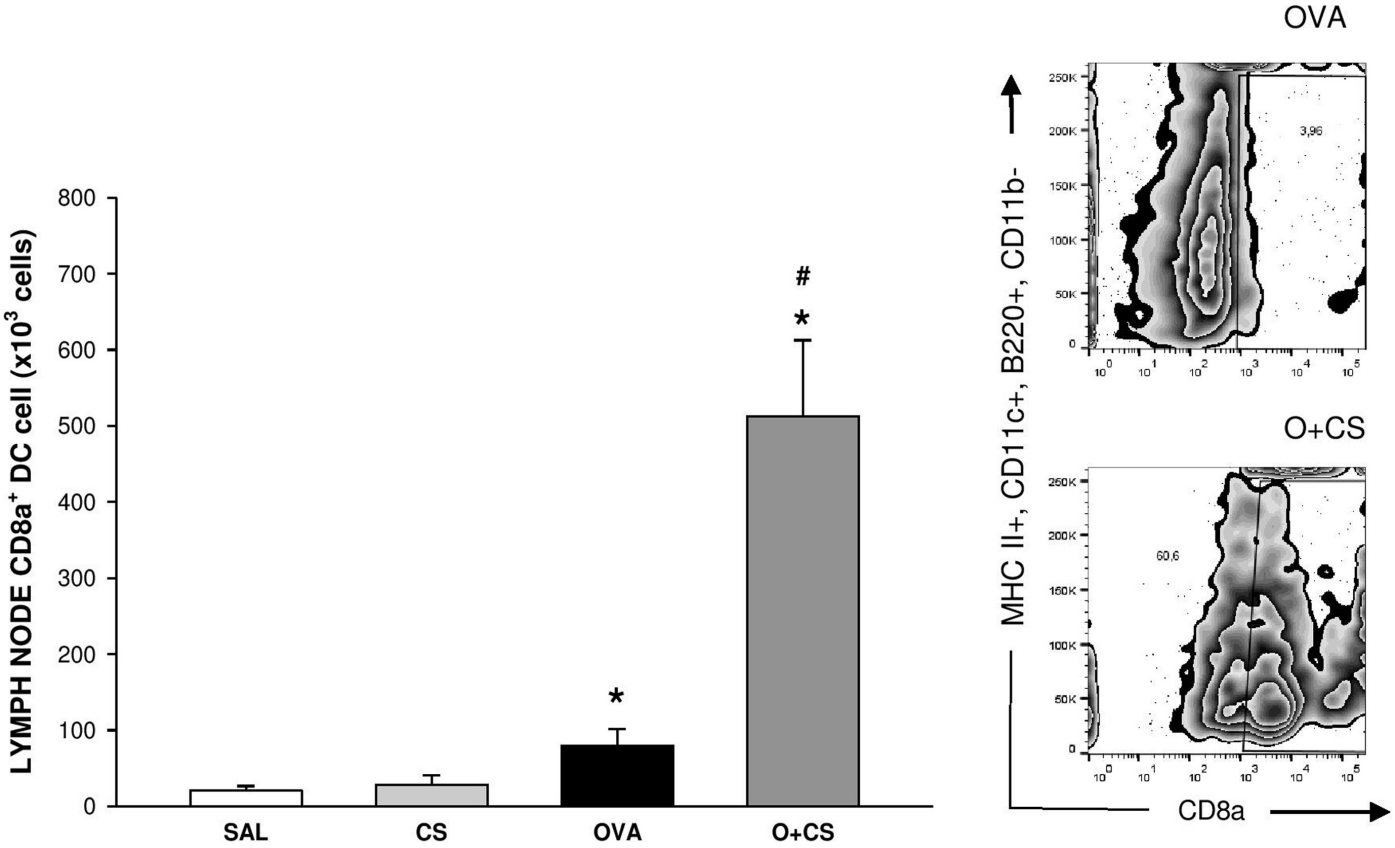
Number of CD8α^+^ dendritic cells (DCs) in lymph nodes. Results are presented as mean ± SE; *n* = 8 mice/group; *p = 0.043 when compared ovalbumin (OVA) versus SAL and *p* < 0.001 when compared O + CS versus CS; ^#^*p* < 0.001 when compared O + CS versus OVA.

Dendritic cells are the most professional antigen-presenting cells, and they are capable of influencing or determining the immune response to any assault (27). CS exposition trends to increase the number of CD11b^+^ DCs in the lung, but with no significance. OVA sensitization combined with CS exposition decreases significantly CD11b^+^ DCs compared to CS alone (Figure 6A); no difference among groups was observed on activation analysis of CD11b^+^ DCs (data not shown). The number of pDCs increased in the lungs of the OVA group and was significantly reduced in the O + CS group (Figure 6B). Activation determined by CD86^+^, ICOSL^+^, and PDL2^+^ costimulatory molecules expression showed decreased in O + CS group (Figure 6C). We then analyzed lymph node DCs, and we observed an increase in the number of CD8α^+^ DCs in the OVA group. Interestingly, there was a strong and significantly increase in the number of CD8α^+^ DCs in the lymph nodes of the O + CS group (Figure 7).

## Discussion

Ovalbumin sensitization caused extensive pulmonary inflammatory infiltrate, increased bronchial responsiveness to methacholine, production of OVA-specific antibodies such as IgE, increased airway remodeling driven by collagen fibers, enlargement of epithelial cells, mucus production, and increased inflammatory cytokine production in the lungs (23). Cigarette smoke exposure associated with OVA sensitization caused bronchial epithelial cell remodeling, increased the number of neutrophils and activated type 2 macrophages, increased the number and activation of CD4^+^ and CD8^+^ T cells and decreased pDCs and its activation by reducing expression of CD86, ICOSL, and PDL2. Of note, OVA combined with CS also decreased T reg cells and anti-inflammatory cytokines. Moreover, the O + CS group showed no change in IgE levels compared to the OVA group. Corroborating with our findings, a study with OVA sensitization and challenge but prolonged CS exposure showed similar results, suggesting that, in this type of model, CS is not able to increase antibody production more than that already seen in allergic animals (23).

Experimental trials showed the aggressive role of CS in asthma with increased remodeling in lung of allergic mice exposed to CS (28, 29). We observed a decrease of eosinophils in BAL accompanied by a significant increase in neutrophils in lungs in the O + CS group. CS acts recruiting neutrophils to the airways of asthmatic subjects and, therefore, worsens the response to corticosteroid treatment and also adds the Th1 response leading to lower Th2 response but with mixed phenotype (30, 31). Smoking plus asthma is also characterized by increased levels of macrophages that lead to greater small airway remodeling (32). We found, in the O + CS group, a drastic increase in the number of alveolar macrophages (M2) that were generally recruited in allergic processes and directly involved with tissue remodeling, here connecting the data already described. We also found a small number of type 1 macrophages (M1), which can lead to susceptibility to infection (26). Despite the increased number of CD4^+^ T cells in lungs, we found reduced levels of IL-4 and IL-13 in O + CS mice. Mice that lack key Th2 cytokines such IL-4 or IL-13 had substantial reductions in asthma features in the OVA model. IL-4 is necessary for the development of adaptive Th2 immunity and IgE antibodies to OVA (33). In this study, despite the decrease in IL-4 levels in O + CS group, we observed that IgE levels were not affected in this group. IL-13 was found to contribute to increased airway responsiveness and goblet cell metaplasia (33). In humans, blocking receptors for IL-4 and IL-13 improves lung function and reduces the frequency of exacerbations in individuals with moderate-to-severe asthma with high levels of eosinophils (34). CS seems to have an immunomodulatory effect in asthma by inhibiting the release of proinflammatory cytokines from macrophages (35). The reduction in IL-4 and IL-13 might indicate that CS altered the Th1/Th2 balance in the allergic response.

However, beyond the reduced proinflammatory cytokine levels, we also found a decrease in anti-inflammatory cytokines such as IL-10 and TGF-β in the O + CS group. TGF-β is a pleiotropic cytokine that is known to have immunosuppressive effects (36), but some studies suggest that CS stimulates the increased production of TGF-β, exacerbating airway inflammation and increasing remodeling in asthma (37). Here, we could see that the reduction in TGF-β did not decrease airway remodeling. IL-10, in turn, has a regulatory and an anti-inflammatory function, and it inhibits antigen presentation in macrophages/monocytes in asthma disease (38). IL-10 knockout mice do not develop airway hyper responsiveness after allergen sensitization and challenge, despite a significant pulmonary inflammatory response that includes increased airway eosinophilia (39). Moreover, we found a decrease in the number of T reg cells, related to the reduced levels of IL-4 and IL-10, as described above. These cytokines stimulate DCs, mostly pDCs, which will polarize naïve T cells to become T regs. Activated T regs produce and release more TGF-β, which activates more pDCs, leading to a loop system (40).

We show for the first time that CS combined with OVA sensitization and challenge promoted significant increase in the number of CD8α^+^ DCs in mediastinal lymph nodes as well as an increased number of CD8^+^ T cells in the lung and increased CD8^+^ T cell activation, as indicated by increased CD69 expression. CD8α^+^ DCs enhance cross-priming of CD8^+^ T cells redirecting the immune response in asthma away from Th2 response (41), as shown here with decrements of IL-4 and IL-13 levels, decreased number of eosinophils. This cell type has been described in respiratory antiviral response (42), but its role in asthma-COPD overlap is unclear. Activated CD8^+^ T cells increase inflammatory cell apoptosis, as studies corroborating with ours, have observed a reduction in inflammatory cells and consequently the cytokines released by them (43, 44). It is important to note that different results may occur in different studies due to the different types of exposure, different concentrations of cigarette smoke, and different asthma phenotypes (45).

With this study, we demonstrated that there is more than one pathway to regulate allergic airway inflammation caused by allergen and CS exposure associated. We suggest that, in late stages of respiratory allergic inflammation, the reduction of pDCs and the increased numbers of CD8α + DCs DCs play a role together in changing Th2 response when there is a detrimental effect of chronic cigarette smoke exposure. The increase of CD8α + DCs by cigarette smoke exposition and allergen challenge is related to lower Th2 inflammation, here and demonstrated with reductions in IL-4 and IL-13 levels. Despite the reduction of Th2 immune response, we could still see the damage caused by CS in the irreversible increase in tissue remodeling and the increased number of proapoptotic cells and their activation in allergic mice. We believe that further studies are necessary to clarify how CS acts in the regulation of asthma in order to improve treatments for this disease.

## Author Contributions

TB performed the experiments, analyzed all data, and drafted the manuscript text. PF helped with performing the experiments. LO and MS helped planning the flow cytometry experiments and LO performed it. MM helped reviewing the data and helped to write the manuscript. FA-C designed the study, reviewed data, and helped to write the manuscript text.

## Conflict of Interest Statement

The authors declare that the research was conducted in the absence of any commercial or financial relationships that could be construed as a potential conflict of interest.
