# Supplementary material for: Cigarette Smoke Increases CD8α+ Dendritic Cells in an Ovalbumin-Induced Airway Inflammation
Source: Front Immunol. 2017 Jun 16;8:718. doi: 10.3389/fimmu.2017.00718 (PMC5472682; doi:10.3389/fimmu.2017.00718)
Supplement: Supplementary file 1 [file data_sheet_1.pdf]

Supplementary Material

Cigarette Smoke Increases CD8α+ Dendritic Cells in an OVA-induced airway inflammation

Thayse Regina Brüggemann, Paula Fernandes, Luana de Mendonça Oliveira, Maria Notomi Sato, Mílton de Arruda Martins, Fernanda Magalhães Arantes-Costa*

*** Correspondence:** Fernanda Magalhães Arantes Costa: fernanda.arantes@fm.usp.br

1 Supplementary Figures

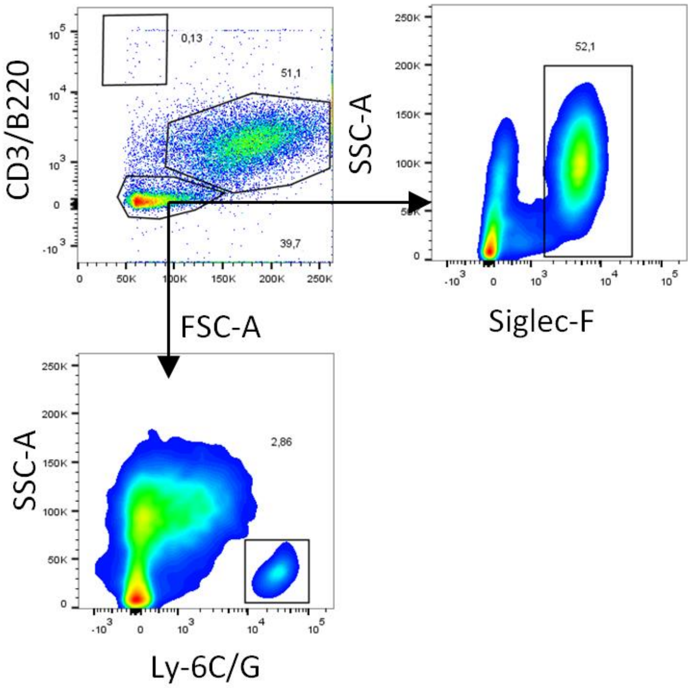

Supplementary Figure 1. Analysis strategy for eosinophils in bronchoalveolar lavage fluid (BALF) and neutrophils in lung. Granulocytes are considered FSC-A low and CD3/B220 negative. Then, eosinophils were considered and Siglec-F positive and neutrophils were considered Ly-6C/G high.

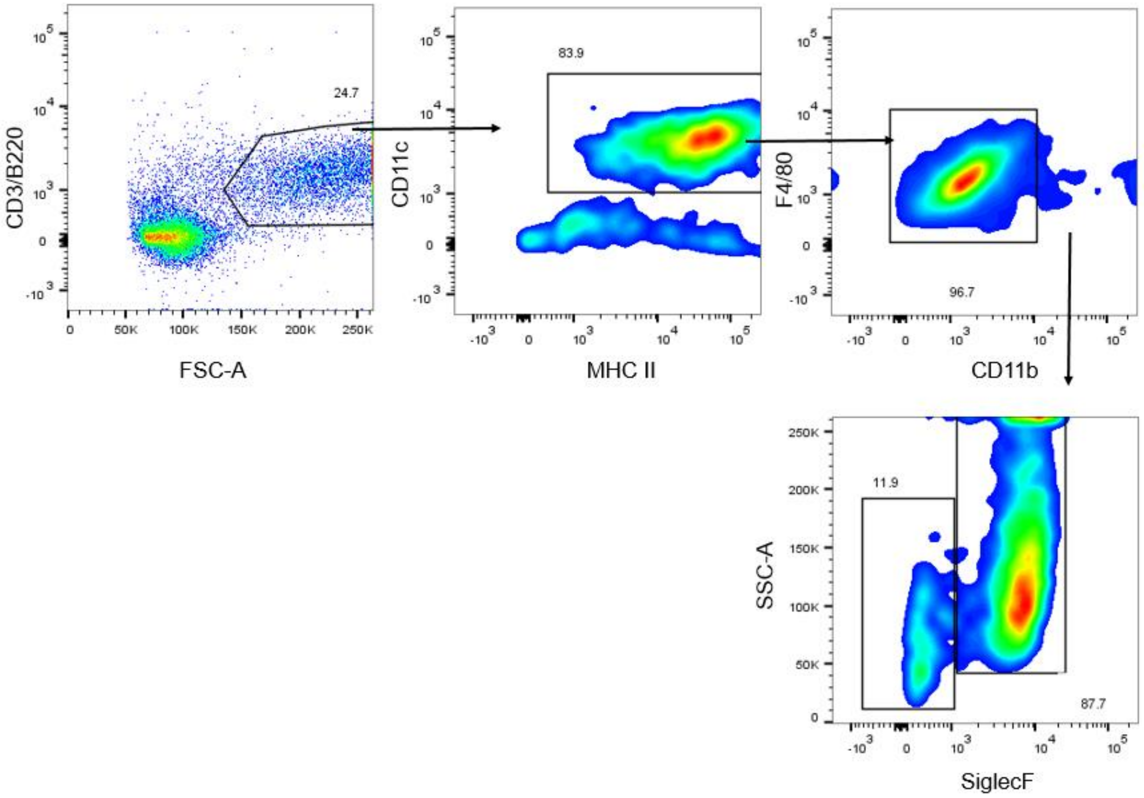

Supplementary Figure 2. Analysis strategy for types 1 and 2 macrophages in bronchoalveolar lavage fluid (BALF). Macrophages are considered FSC-A high and CD3/B220 intermediate/low. Type 1 macrophages were considered CD11c positive, MHC II positive and Siglec-F negative. Type 2 macrophages were considered CD11c positive, MHC II postivie and Siglec-F positive.

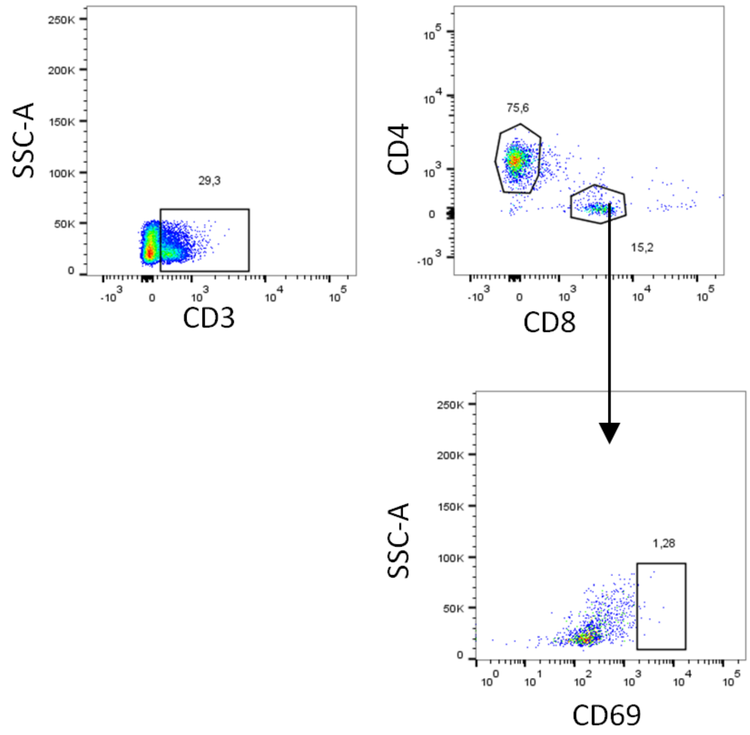

Supplementary Figure 3. Analysis strategy for $CD4^+$ and $CD8^+$ T lymphocytes and activation of $CD8^+$ T lymphocytes by expression of CD69 in lung. All lymphocytes were considered positive for CD3. $CD8^+$ T lymphocytes were considered activated when were positive for CD69.

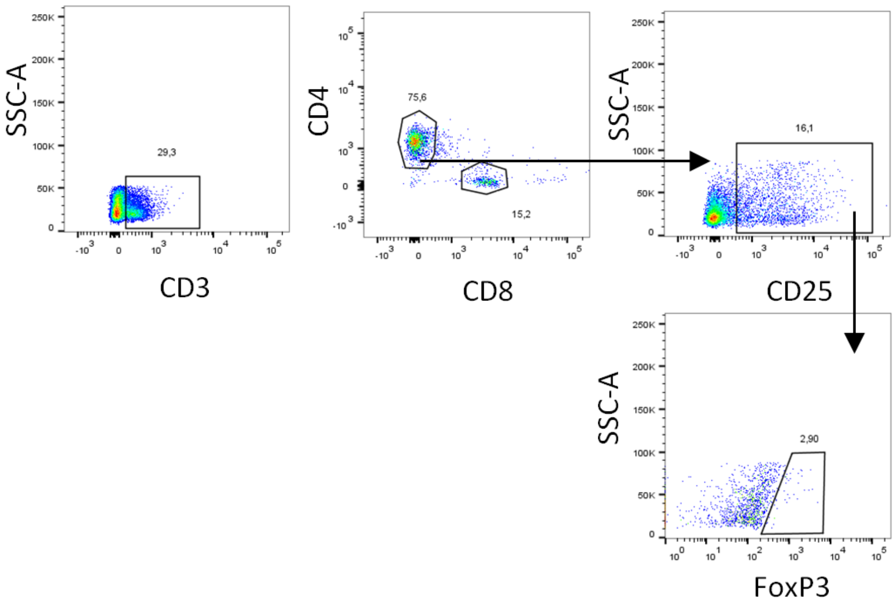

Supplementary Figure 4. Analysis strategy for regulatory T cells (T regs) in lung. T regs were considered CD3, CD4, CD25 and Foxp3 positive.

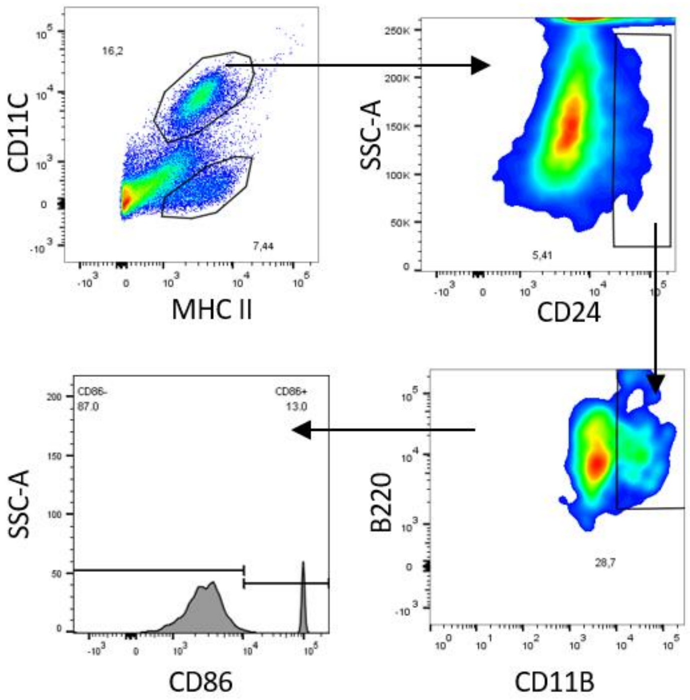

Supplementary Figure 5. Analysis strategy for CD11b+ dendritic cells (CD11b+ DCs) and activation by expression of CD86 in lung. CD11b+ DCs were considered CD11c and MHC II high, positive for CD24, B220 and CD11b high. Cells were considered activated when positive for CD86.

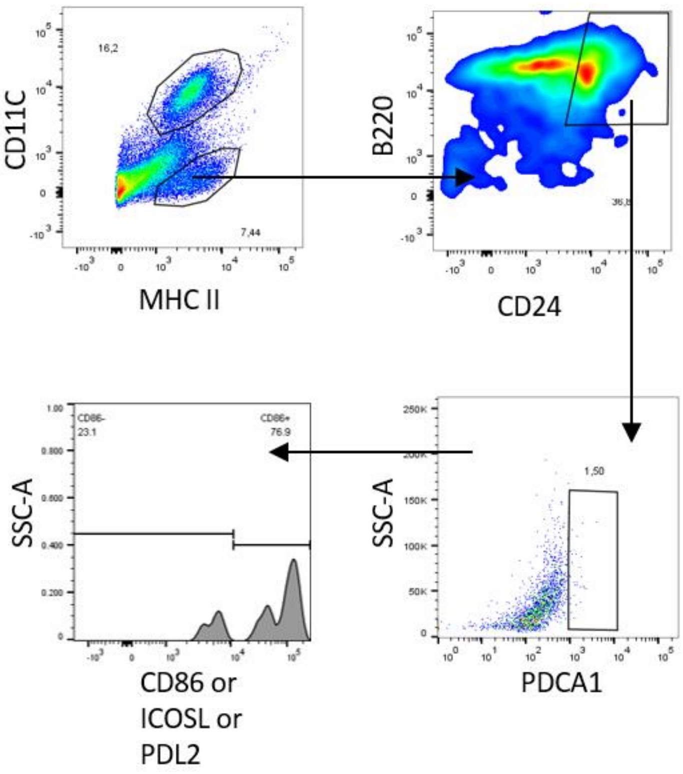

Supplementary Figure 6. Analysis strategy for plasmacytoid DCs (pDCs) and activation by expression of CD86, ICOSL and PDL2 in lung. pDCs were considered CD11c negative or low, MHC II low, positive for B220, CD24 and PDCA1. Cells were considered activated when positive for CD86, or ICOSL or PDL2.

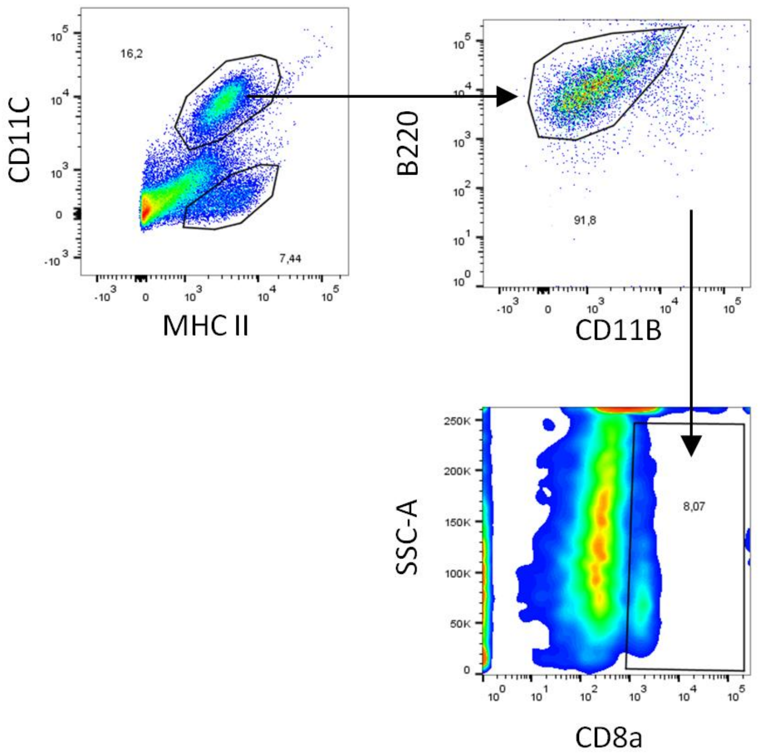

Supplementary Figure 7. Analysis strategy for CD8α+ DCs in lymph node. CD8α+ DCs were considered CD11c and MHC II high, B220 positive, CD11b negative and CD8a positive.
